# Hyperglycemia-Induced Oxidative Stress Abrogates Remifentanil Preconditioning-Mediated Cardioprotection in Diabetic Rats by Impairing Caveolin-3-Modulated PI3K/Akt and JAK2/STAT3 Signaling

**DOI:** 10.1155/2019/9836302

**Published:** 2019-09-05

**Authors:** Shaoqing Lei, Wating Su, Zhong-Yuan Xia, Yafeng Wang, Lu Zhou, Shigang Qiao, Bo Zhao, Zhengyuan Xia, Michael G. Irwin

**Affiliations:** ^1^Department of Anesthesiology, Renmin Hospital of Wuhan University, Wuhan, China; ^2^Department of Anesthesiology, University of Hong Kong, Hong Kong SAR, China; ^3^State Key Laboratory of Pharmaceutical Biotechnology, University of Hong Kong, Hong Kong SAR, China

## Abstract

Diabetic hearts are more vulnerable to ischemia/reperfusion (I/R) injury and less responsive to remifentanil preconditioning (RPC), but the underlying mechanisms are incompletely understood. Caveolin-3 (Cav-3), the dominant isoform of cardiomyocyte caveolae, is reduced in diabetic hearts in which oxidative stress is increased. This study determined whether the compromised RPC in diabetes was an independent manifestation of hyperglycemia-induced oxidative stress or linked to impaired Cav-3 expression with associated signaling abnormality. RPC significantly attenuated postischemic infarction, cardiac dysfunction, myocardial apoptosis, and 15-F2t-isoprostane production (a specific marker of oxidative stress), accompanied with increased Cav-3 expression and enhanced Akt and STAT3 activation in control but not in diabetic rats. Pretreatment with the antioxidant N-acetylcysteine (NAC) attenuated hyperglycemia-induced reduction of Cav-3 expression and Akt and STAT3 activation and restored RPC-mediated cardioprotection in diabetes, which was abolished by cardiac-specific knockdown of Cav-3 by AAV9-shRNA-Cav-3, PI3K/Akt inhibitor wortmannin, or JAK2/STAT3 inhibitor AG490, respectively. Similarly, NAC could restore RPC protection from high glucose and hypoxia/reoxygenation-induced injury evidenced by decreased levels of LDH release, 15-F2t-isoprostane, O_2_^−^, and JC-1 monomeric cells, which were reversed by caveolae disrupter methyl-*β*-cyclodextrin, wortmannin, or AG490 in isolated primary cardiomyocytes or siRNAs of Cav-3, Akt, or STAT3 in H9C2 cells. Either methyl-*β*-cyclodextrin or Cav-3 knockdown reduced Akt and STAT3 activation. Further, the inhibition of Akt activation by a selective inhibitor or siRNA reduced STAT3 activation and vice versa, but they had no effects on Cav-3 expression. Thus, hyperglycemia-induced oxidative stress abrogates RPC cardioprotection by impairing Cav-3-modulated PI3K/Akt and JAK2/STAT3 signaling. Antioxidant treatment with NAC could restore RPC-induced cardioprotection in diabetes by improving Cav-3-dependent Akt and STAT3 activation and by facilitating the cross talk between PI3K/Akt and JAK2/STAT3 signaling pathways.

## 1. Introduction

Ischemic heart disease (IHD) is a leading cause of heart failure and death in patients with diabetes mellitus worldwide [1]. Hyperglycemia-induced oxidative stress plays a vital role in the development and progression of IHD [2, 3]. Prompt restoration of blood flow to the ischemic tissue is a standard clinical therapy in myocardial infarction, but paradoxically, this may cause oxidative cell injury and additional cell death called “ischemia-reperfusion (I/R) injury” [4, 5]. Hearts of diabetic subjects are more vulnerable to I/R injury [6] and less responsive to many therapeutic strategies (e.g., ischemic conditioning [6–8] and opioid conditioning [9, 10]), which are effective in protecting against myocardial I/R injury in nondiabetic conditions [11, 12]. Of note, ischemic preconditioning-induced cardioprotection can be abolished by opioid receptor (OR) antagonists [13] and mimicked by OR agonists (e.g., remifentanil) preconditioning [12], suggesting the involvement of some similar cellular mechanisms in cardioprotection by both ischemic preconditioning and opioid preconditioning. Remifentanil is an ultrashort-acting, potent opioid analgesic that is widely used during surgery. Remifentanil preconditioning (RPC) has been reported to reduce myocardial I/R injury in normal conditions [12, 14], but its effectiveness is compromised under hyperglycemia [10, 15]. However, the underlying mechanisms by which hyperglycemia compromises RPC cardioprotection in diabetic hearts have not been elucidated.

It is generally considered that the classic ischemic conditioning-mediated cardioprotection is associated with the activation of the reperfusion injury salvage kinase (RISK) pathway (i.e., phosphatidylinositol 3-kinase (PI3K)/Akt) and survivor activating factor enhancement (SAFE) pathway (i.e., Janus-activated kinase-2 (JAK2)/signal transducer and activator of transcription-3 (STAT3)) [16, 17]. A previous study has shown that opioid-induced cardioprotection occurs via the PI3K pathway-dependent Akt and JAK2 regulation of STAT3 activation [18]. RPC can enhance the cell viability and maturation of osteoblasts by enhancing Akt phosphorylation [19], and Akt activation is also a mechanism of RPC in isolated neonatal rat ventricular myocytes [15]. Furthermore, our recent study shows that RPC can attenuate postischemic myocardial infarction and cardiac dysfunction through activation of JAK2/STAT3 signaling in normal rats [12]. All these findings suggest that both PI3K/Akt and JAK2/STAT3 signaling pathways play important roles in RPC cardioprotection. However, little is known about their relationship and relative roles in RPC cardioprotection in diabetes.

Caveolae, lipid-rich microdomains of the sarcolemma, serve as a platform to localize and enrich cardioprotective signaling molecules and modulate transduction pathways via signaling molecules docked within caveolins. Caveolin-3 (Cav-3), the dominant isoform in cardiac myocytes, is a determinant of caveolae formation [20]. Increasing evidences indicate that Cav-3 is necessary for the myocardium to retain tolerance to I/R injury [21, 22] and is required for opioid preconditioning to confer cardiac protection [23]. However, in diabetes, the cardiac Cav-3 expression is impaired by hyperglycemia-induced oxidative stress [24], accompanied with lower activation of Akt and STAT3 [25, 26], while the diabetic hearts are more vulnerable to I/R injury [25, 27] and less responsive to RPC [10, 15]. However, it remains unclear whether the compromised RPC cardioprotection in diabetes is an independent manifestation of hyperglycemia-induced oxidative stress or linked to impaired Cav-3 expression and PI3K/Akt and JAK2/STAT3 signaling.

In this study, we hypothesize that hyperglycemia-induced oxidative stress compromises RPC cardioprotection due to caveola dysfunction with associated signaling abnormality. Our data suggest that the inhibition of excessive oxidative stress by N-acetylcysteine (NAC), an antioxidant which has been proven by us [28, 29] and others [30] to attenuate myocardial I/R injury in diabetes, could restore Cav-3 expression, which subsequently improves PI3K/Akt and JAK2/STAT3 signaling and ultimately restores RPC cardioprotection in diabetic rats.

## 2. Methods

### 2.1. Experimental Animals and Induction of Diabetes

Male Sprague-Dawley rats (250 ± 10 g, 6-8 weeks) were obtained from the Laboratory Animal Service Center of Wuhan University. The Institutional Animal Care and Use Committee approved this animal experimentation, which meets the *International Guiding Principles for Biomedical Research Involving Animals*, as issued by the Council for International Organizations of Medical Sciences. Diabetes was induced by a single tail vein injection of streptozotocin (STZ, Sigma, St. Louis, MO, USA) at a dose of 60 mg/kg body weight in 0.1 mmol/L citrate buffer under halothane anesthesia, while control rats were injected with equal volume of citrate buffer alone. After 72 hours (h) of injection, the rats with fasting plasma glucose levels ≥ 16.7 mmol/L at least three times were considered as diabetes. One week after induction of diabetes, rats were treated by oral gavage with vehicle or NAC (Sigma, St. Louis, MO, USA) for 4 weeks at the dose of 1.5 g kg^−1^ day^−1^ as described previously [31]. At the end of the study, fasting plasma insulin and triglycerides were measured by the corresponding assay kits (Nanjing Jiancheng Bioengineering Institute, China).

### 2.2. AAV (Adeno-Associated Virus) Infection In Vivo

After NAC treatment for 1 week, a subgroup of diabetic rats were administrated with AAV9-GFP (control) or AAV9-Cav-3 shRNA-GFP (Hanbio Biotechnology Co., Shanghai, China) via a single tail vein injection at a dose of 1 × 10^12^ vg/kg according to the manufacturer's instructions. The rats continue to be treated with NAC for 3 weeks, and then the rats were subjected to myocardial I/R insult as described below.

### 2.3. Myocardial I/R Injury Model

**Figure 1:**
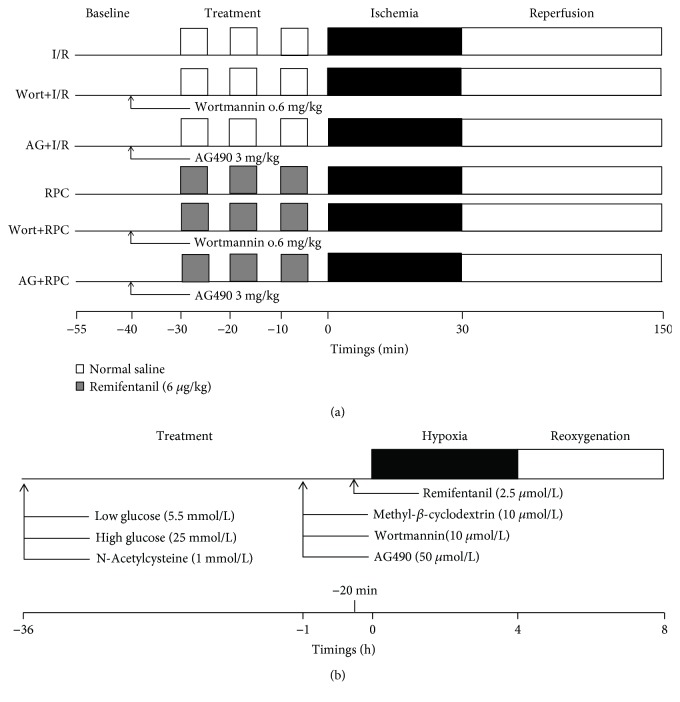
Schematic illustration of experimental protocols used to explore the compromised cardioprotection of remifentanil preconditioning (RPC) in diabetes in *in vivo* (a) and *in vitro* (b) studies. I/R: ischemia/reperfusion; Wort: wortmannin; AG: AG490.

Myocardial I/R injury was achieved by occluding the left anterior descending (LAD) coronary artery for 30 min followed by reperfusion for 2 h as we described [6, 27]. Rats in the sham group underwent the same surgical procedures without ligation. A single dose of remifentanil (6 *μ*g/kg/min) (GlaxoSmithKline, Kowloon, Hong Kong, China) was administered according to our previous study [12], in which remifentanil was given intravenously before ischemia by 3 consecutive 5 min infusions interspersed with 5 min infusion-free periods, termed as RPC. A subgroup of rats were, respectively, given the putative PI3K inhibitor wortmannin (Wort, 0.6 mg/kg [6]) (Sigma, St. Louis, MO, USA) and JAK2 inhibitor AG490 (AG, 3 mg/kg [18]) (Sigma, St. Louis, MO, USA) 10 min before remifentanil administration (Figure 1(a)). During the process of ischemia and reperfusion, hemodynamic changes of the experimental rats were monitored as we previously described [6]. The heart rate (HR), left ventricular systolic pressure (LVSP), maximum rate of increase of left ventricular developed pressure (+dp/dt), and maximum rate of decrease of left ventricular developed pressure (-dp/dt) were recorded at 10 min before ischemia (baseline) and 2 h after reperfusion. At the end of reperfusion, carotid artery blood and ischemic heart tissue samples were collected and stored at -80°C for further analysis.

### 2.4. Determination of the Myocardial Infarct Size

At the end of 2 h reperfusion, myocardial infarct size of the experimental rats was measured using Evans blue dye/2,3,5-triphenyltetrazolium chloride (TTC, Sigma, St. Louis, MO, USA) staining as we previously described [6]. The unstained region by Evans blue dye was considered as the area at risk (AAR). The area unstained by TTC was identified as the infarcted tissue. The volumes of the left ventricles (LV), infarcted area, and AAR were calculated by multiplying each area with slice thickness and summing the product. Myocardial infarct size was expressed as a percentage of the AAR (% AAR).

### 2.5. Measurement of CK-MB and 15-F2t-isoprostane

Plasma samples were separated from the collected carotid artery blood at the end of 2 h reperfusion. Plasma CK-MB (creatine kinase-MB), a major biomarker of myocardial I/R injury, was measured using a commercial ELISA kit (Elabscience Biotechnology, Wuhan, China) according to the manufacturer's instructions as we described [6, 32]. 15-F2t-isoprostane (15-F2t-IsoP), a specific marker of oxidative stress [33], was measured using an EIA kit (Cayman Chemical, Ann Arbor, MI, USA) as described previously [31]. The values of 15-F2t-IsoP in plasma and cultured medium were expressed as pg/mL, and the values of 15-F2t-IsoP in homogenized ischemic heart tissues were expressed as pg/mg protein.

### 2.6. Apoptosis Assay

At the end of 2 h reperfusion, the left ventricular apex (ischemic heart tissues) was harvested immediately after the sacrifice and then designed to become paraffin-embedded sections. Myocardial apoptosis was measured using TUNEL (terminal deoxynucleotidyl transferase dUTP nick-end labeling) with an *in situ* cell death detection kit (Roche Applied Science, Mannheim, Germany) according to the manufacturer's instructions as we described previously [6]. TUNEL-positive cells displayed the nuclei with brown staining. Ten different fields in each slide were randomly selected and blindly analyzed. The apoptotic index was calculated as a percentage of TUNEL-positive cells to total cells, which was used to evaluate the apoptotic status.

### 2.7. Preparation of Isolated Rat Ventricular Cardiomyocytes

A modified method was used to isolate calcium-tolerant cardiomyocytes from adult rat ventricles, as we described previously [26]. Isolated primary cardiomyocytes from a single rat heart were plated on Matrigel-coated culture dishes and allowed to recover for 3 h. Then, the cardiomyocytes were incubated in low glucose (LG, 5.5 mmol/L), mannitol/glucose (19.5 mmol/L mannitol plus 5.5 mmol/L glucose), or high glucose (HG, 25 mmol/L) with or without treatment of methyl-*β*-cyclodextrin (CD, 10 *μ*mol/L, a disrupter of cholesterol-rich caveolae [34]), wortmannin (Wort, 100 nmol/L [6, 12]), AG490 (AG, 50 *μ*mol/L [6]), or combination of remifentanil (2.5 *μ*mol/L [12]) and N-acetylcysteine (NAC, 1 mmol/L [29]) for 36 h as we described previously [26], in which high glucose duration for 36 h significantly induced cardiomyocyte injury and impaired Cav-3 expression. NAC was added to the cultured medium at the same time as HG exposure. CD, Wort, and AG were given 1 h before hypoxia stimulation, and remifentanil was administered 20 min before inducing hypoxia (Figure 1(b)). Hypoxia/reoxygenation (H/R) induction was achieved by exposing the cardiomyocytes to 4 h of hypoxia (1% O_2_, 5% CO_2_, and 94% N_2_) followed by 4 h of reoxygenation, as described previously [27].

### 2.8. Small Interfering RNA (siRNA) Studies in H9C2 Cells

Embryonic rat cardiomyocyte-derived H9C2 cells were maintained in Dulbecco's modified Eagle's medium (DMEM) containing 10% FBS in a humidified atmosphere (5% CO_2_) at 37°C. Commercial Cav-3 siRNA, Akt siRNA, and STAT3 siRNA (Santa Cruz Biotechnology) were used for inhibiting Cav-3, Akt, and STAT3 protein expression, respectively, according to the manufacturer's instructions. After transfection with control, Cav-3 siRNA, Akt siRNA, or STAT3 siRNA, cells were treated with N-acetylcysteine (NAC, 1 mmol/L [29]) or combination with remifentanil (2.5 *μ*M [12]) in HG condition for 36 h; then, the cells were exposed to H/R stimulation.

### 2.9. Cell Viability and LDH (Lactate Dehydrogenase) Release

Cell viability and LDH release were measured to evaluate the extent of cellular injury in primary cardiomyocytes and H9C2 cells using CCK-8 and LDH assay kits, respectively (Nanjing Jiancheng Bioengineering Institute, China) according to the manufacturer's instructions.

### 2.10. Determination of O_2_^−^ Production

The levels of O_2_^−^ production were assayed using the lucigenin chemiluminescence method [35, 36]. The protein concentration of the samples was measured with a Bio-Rad Protein Assay kit. The samples were loaded with dark-adapted lucigenin (5 *μ*M) and read in 96-well microplates using a luminometer (GloMax, Promega, USA). Light emission was recorded for 5 min and expressed as mean light units (MLU)/min/100 *μ*g protein.

### 2.11. Mitochondrial Membrane Potential Assessment

Mitochondrial membrane potential (MMP) was assessed using a JC-1 MMP ELISA kit (Cayman Chemical, Ann Arbor, MI, USA) as we reported to evaluate mitochondrial damage [6, 12]. Red fluorescence, which appears in the matrix of actively respiring mitochondria, is the aggregate form of JC-1 molecules. Green fluorescence is emitted from the monomeric form of the JC-1 molecules, which is formed after mitochondrial membrane depolarization. The fluorescence was measured using a luminometer (GloMax, Promega, USA), and the loss of MMP was assessed by the percentage of green cells in total cells. In apoptotic cells, the loss of MMP results in the formation of JC-1 monomeric mitochondria.

### 2.12. Western Blot Analysis

Equal amounts of protein from the myocardial tissue or cell lysate were separated via 7.5-12.5% SDS-PAGE and subsequently transferred to the PVDF membrane for immunoblot analysis as described previously [31]. Primary antibodies against Cav-3 (1 : 500, Santa Cruz Biotechnology), Akt (1 : 1000, Cell Signaling Technology), phosphorylated (p)-Akt at ser^473^ (1 : 500, Cell Signaling Technology), STAT3 (1 : 1000, Cell Signaling Technology), p-STAT3 at Tyr^705^ and ser^727^ (1 : 500, Cell Signaling Technology), and GAPDH (1 : 2000, Cell Signaling Technology) were used in the present study. The results were normalized to GAPDH to correct for loading. Data are presented as percent change relative to the control measurement.

### 2.13. Statistical Analysis

All the values are expressed as means ± S.D. A GraphPad Prism software program (GraphPad Software Inc., San Diego, CA, USA) was used for statistical analysis. Comparison between multiple groups was made with one-way analysis of variance (ANOVA) followed by Tukey's test for multiple comparisons or two-way repeated-measures ANOVA followed by Bonferroni's post hoc test in grouped values. *P* < 0.05 was considered statistically significant.

## 3. Results

### 3.1. RPC Attenuated Myocardial I/R Injury in Control but Not in Diabetic Rats

**Figure 2:**
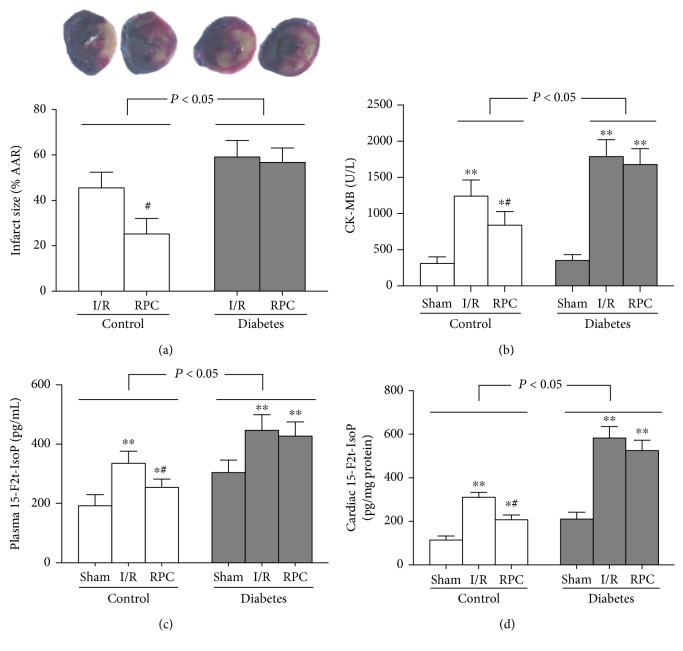
Remifentanil preconditioning (RPC) attenuated myocardial I/R injury in control but not in diabetic rats. Control or streptozotocin-induced diabetic rats were subjected to sham operation, myocardial ischemia/reperfusion (I/R), or RPC. Infarct size (a), plasma CK-MB (b), plasma 15-F2t-isoprostane (15-F2t-IsoP) (c), and cardiac 15-F2t-IsoP (d). All the results are expressed as means ± S.D., *n* = 7. Differences were analyzed by two-way repeated-measures ANOVA followed by Bonferroni's post hoc test. ^∗^*P* < 0.05 and ^∗∗^*P* < 0.01 vs. the corresponding sham group; ^#^*P* < 0.05 vs. the corresponding I/R group.

At the end of this study (5 weeks after the onset of induction of diabetes), the diabetic rats had higher water intake, food consumption, plasma glucose, and plasma triglycerides and lower body weight and plasma insulin than control rats (Table 1). When the rats underwent myocardial I/R, the infarct size (% AAR) in diabetic rats (including I/R and RPC groups) was larger than that in the corresponding control rats (Figure 2(a)), though there was no significant difference in AAR/LV among the various groups (data not shown). RPC significantly attenuated the infarct size in control rats but failed to elicit similar effects in diabetic rats (Figure 2(a)), indicating that RPC-mediated cardioprotection was compromised by diabetes. We then measured the biochemical markers of myocardial I/R injury and oxidative stress in the experimental rats. As shown in Figures 2(b)–2(d), both the plasma and cardiac levels of 15-F2t-IsoP in diabetic rats subjected to sham operation, I/R, and RPC were significantly higher than those in the corresponding nondiabetic control rats, as well as higher plasma CK-MB in diabetic rats which underwent I/R and RPC. Myocardial I/R significantly increased the levels of plasma CK-MB, plasma 15-F2t-IsoP, and cardiac 15-F2t-IsoP as compared with the corresponding sham rats. RPC significantly attenuated the increases of plasma CK-MB and plasma and cardiac 15-F2t-IsoP induced by myocardial I/R in control but not in diabetic rats (Figures 2(b)–2(d)).

### 3.2. RPC Increased Cav-3 Expression and Enhanced Akt and STAT Activation in Control but Not in Diabetic Rats

**Figure 3:**
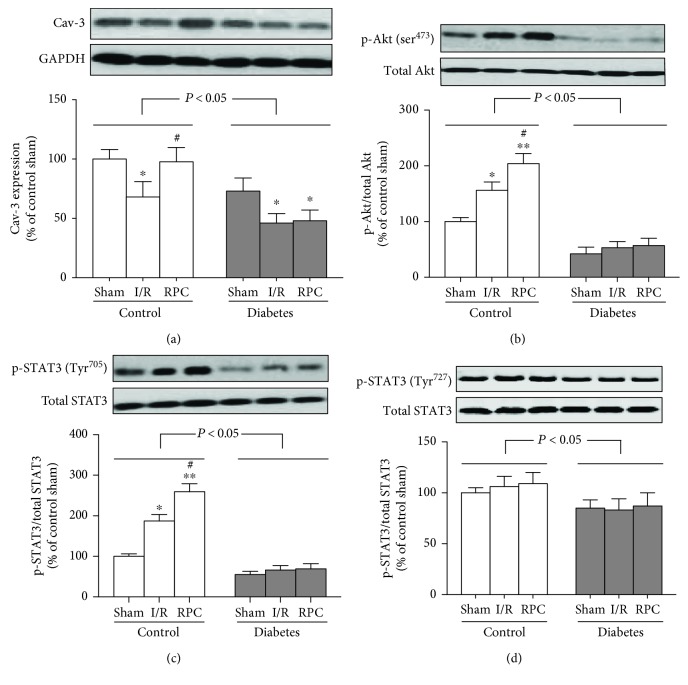
RPC improved Cav-3 expression and enhanced Akt and STAT activation in control rats but not in diabetic rats. Control or streptozotocin-induced diabetic rats were subjected to sham operation, myocardial ischemia/reperfusion (I/R), or RPC. (a–d) Representative Western blot of Cav-3 compared with GADPH (a), p-Akt (ser^473^) compared with total Akt (b), p-STAT3 (Tyr^705^) (c), and p-STAT3 (Tyr^727^) (d) compared with total STAT3. All the results are expressed as mean ± S.D., *n* = 7. Differences were analyzed by two-way repeated-measures ANOVA followed by Bonferroni's post hoc test. ^∗^*P* < 0.05 and ^∗∗^*P* < 0.01 vs. the corresponding sham group; ^#^*P* < 0.05 vs. the corresponding I/R group.

We previously demonstrated impaired Cav-3 expression (a dominant constituent protein of cardiomyocyte caveolae formation [20]) and reduced phosphorylation of Akt and STAT3 in diabetic hearts [25, 26], which was attributable to decreased tolerance to myocardial I/R injury in diabetes [25, 27]. In the present study, we determined the effects of RPC on the cardiac expression of Cav-3, Akt, and STAT3, as well as Akt and STAT3 phosphorylation in control and diabetic rats. As shown in Figure 3(a), myocardial Cav-3 expression in sham, I/R, and RPC groups in diabetic rats was much lower than that in the corresponding nondiabetic control rats. The cardiac expression of Cav-3 was further significantly reduced after myocardial I/R as compared with the corresponding sham rats in control and diabetic groups. RPC significantly attenuated I/R-induced decrease of Cav-3 expression in control but not in diabetic rats. As shown in Figures 3(b)–3(d), diabetes markedly decreased Akt phosphorylation on ser^473^ and STAT3 phosphorylation on Tyr^705^ but slightly decreased STAT3 phosphorylation on ser^727^, without influencing total Akt and total STAT3 expression at baseline, resulting in a markedly decreased ratio of phosphorylated Akt to total Akt and phosphorylated STAT3 to total STAT3. In comparison with sham rats, myocardial I/R significantly increased Akt phosphorylation on ser^473^ and STAT3 phosphorylation on Tyr^705^ but not on ser^727^, which was further increased by RPC in nondiabetic control rats; however, these alterations were not observed in diabetic rats (Figures 3(b)–3(d)).

### 3.3. NAC Treatment Enabled RPC to Attenuate Postischemic Cardiac Dysfunction and Cardiomyocyte Apoptosis in Diabetic Rats

**Figure 4:**
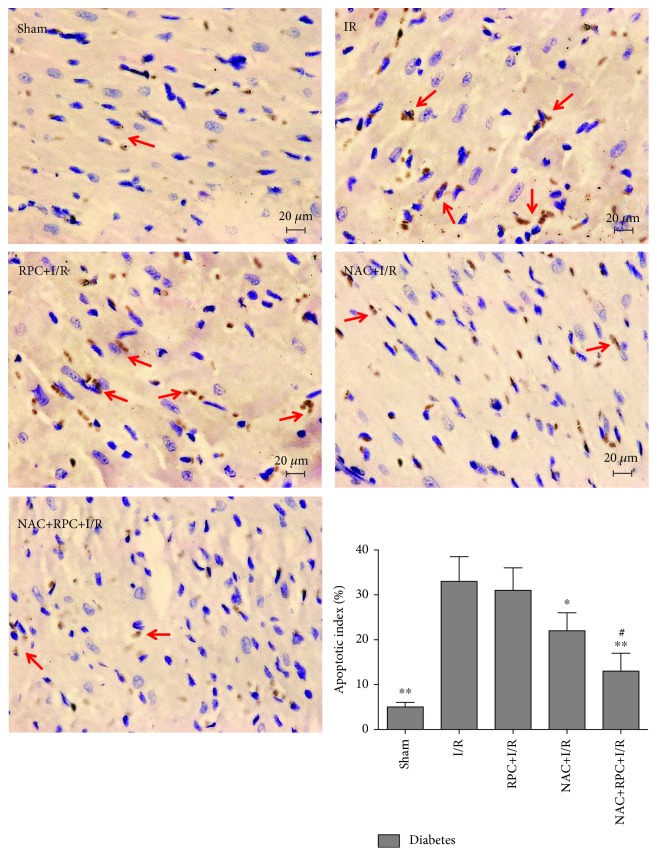
N-Acetylcysteine (NAC) treatment enabled RPC to attenuate cardiomyocyte apoptosis in diabetic rats. Streptozotocin-induced diabetic rats with or without NAC treatment were subjected to sham operation, myocardial ischemia/reperfusion (I/R), or remifentanil preconditioning (RPC). Representative apoptotic cells were labeled with arrows in the images. All the results are expressed as means ± S.D., *n* = 7. Difference was determined by one-way analysis of variance (ANOVA) followed by Tukey's test. ^∗^*P* < 0.05 and ^∗∗^*P* < 0.01 vs. the I/R group; ^#^*P* < 0.05 vs. the I/R+NAC group.

As excessive oxidative stress is a major mechanism of myocardial I/R injury in diabetes [2, 37], we examined the treatment effects of the antioxidant NAC on RPC in diabetic rats. In the present study, we used NAC at a dose of 1.5 g/kg/day for 4 weeks and such dosage was well demonstrated to attenuate diabetes-induced cardiac damage [31, 38] and myocardial I/R injury [28, 29]. At the end of 4 weeks of NAC treatment, the increase in water intake, food consumption, and plasma triglycerides and decrease in body weight in diabetic rats were significantly attenuated, but the elevated plasma glucose and decreased plasma insulin were not altered (Table 1). After all the experimental rats were subjected to myocardial I/R, we monitored hemodynamics at baseline (10 min before ischemia) and after 2 h of reperfusion. As shown in Table 2, the levels of HR, LVSP, +dp/dt, and –dp/dt in diabetic rats at baseline were significantly reduced as compared with control rats, indicating cardiac dysfunction in diabetes. NAC treatment significantly increased the levels of +dp/dt and –dp/dt without affecting HR and LVSP in diabetic rats. After 2 h of reperfusion, the levels of HR, LVSP, +dp/dt, and –dp/dt in all experimental rats were significantly decreased as compared with those at baseline. RPC significantly increased the levels of HR, LVSP, +dp/dt, and –dp/dt at 2 h of reperfusion in control rats but had no significant effects on hemodynamics in untreated diabetic rats. However, after the four-week treatment with NAC in diabetic rats, the levels of HR, LVSP, +dp/dt, and –dp/dt at 2 h of reperfusion were significantly increased, which were further increased by RPC. We also evaluated the treatment effects of NAC on RPC protection against cardiomyocyte apoptosis in diabetic rats. As shown in Figure 4, myocardial I/R significantly elevated the apoptotic index in diabetic rats as compared with the sham group, but this alteration was not affected by RPC. However, this I/R-induced increase of the apoptotic index was significantly reduced by NAC treatment for 4 weeks, which was further reduced by the combined use of RPC and NAC.

### 3.4. NAC Treatment Restored RPC Cardioprotection against Myocardial I/R Injury in Diabetic Rats but Was Abolished by Inhibition of PI3K/Akt or JAK2/STAT3 Signaling

**Figure 5:**
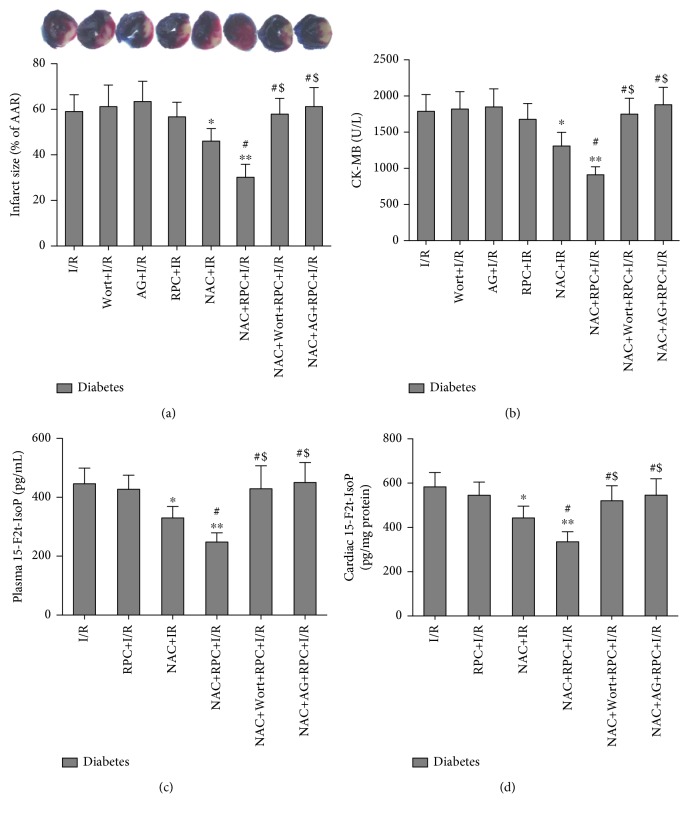
N-Acetylcysteine (NAC) treatment restored remifentanil preconditioning (RPC) in diabetic rats, which was abolished by the inhibition of PI3K/Akt or JAK2/STAT3 signaling. Streptozotocin-induced diabetic rats with or without NAC treatment in the presence or absence of wortmannin (Wort) and AG490 (AG) were subjected to myocardial ischemia/reperfusion (I/R) or RPC. Infarct size (a), plasma CK-MB (b), plasma 15-F2t-isoprostane (15-F2t-IsoP) (c), and cardiac 15-F2t-IsoP (d). All the results are expressed as mean ± S.D., *n* = 7. Differences were determined by one-way analysis of variance (ANOVA) followed by Tukey's test. ^∗^*P* < 0.05 and ^∗∗^*P* < 0.01 vs. the I/R group; ^#^*P* < 0.05 vs. the I/R+NAC group; and ^$^*P* < 0.05 vs. the NAC+RPC group.

We next investigated whether antioxidant NAC could restore RPC cardioprotection in diabetic rats and whether wortmannin and AG490, the putative inhibitor of PI3K/Akt and JAK2/STAT3, respectively, could cancel RPC-mediated cardioprotection in NAC-treated diabetic rats. As shown in Figure 5(a), RPC had no significant effects on the I/R-induced infarct size in untreated diabetic rats. NAC treatment for 4 weeks significantly reduced the postischemic infarct size as compared with untreated diabetic rats, which were further decreased by RPC, indicating that NAC treatment could restore RPC-mediated cardioprotection against myocardial I/R injury in diabetic rats and confer synergistic or added cardioprotection. Although wortmannin or AG490 alone had no significant effects on the infarct size in untreated diabetic rats, RPC-mediated cardioprotection in NAC-treated diabetic rats was abolished by treatment with wortmannin or AG490.

To further confirm the beneficial effects of RPC in NAC-treated diabetic rats, we also determined the levels of CK-MB and 15-F2t-IsoP. As shown in Figures 5(b)–5(d), RPC did not have significant effects on the levels of postischemic plasma CK-MB and plasma and cardiac 15-F2t-IsoP in untreated diabetic rats. With NAC treatment for 4 weeks, the levels of plasma CK-MB, plasma 15-F2t-IsoP, and cardiac 15-F2t-IsoP were significantly decreased as compared with that in untreated diabetic rats sustaining myocardial I/R, which were further decreased by RPC. However, all these protective effects of RPC in NAC-treated diabetic rats were abolished by treatment with wortmannin or AG490.

### 3.5. Effects of RPC on Myocardial Caspase-3 and Cav-3 and the Phosphorylation Status of Akt and STAT3 in NAC-Treated Diabetic Rats with or without Treatment with Wortmannin or AG490

**Figure 6:**
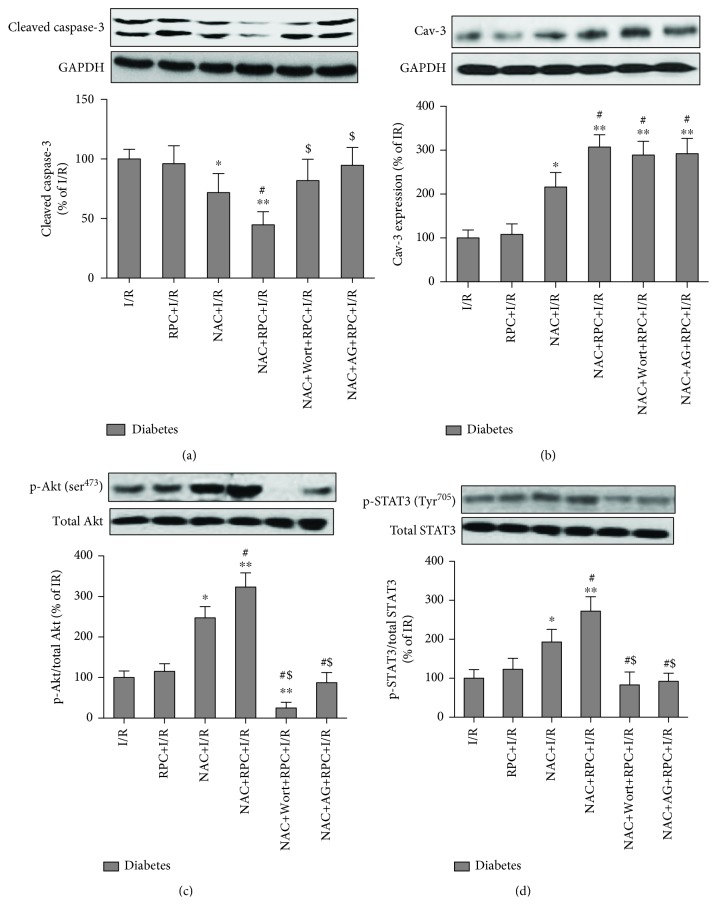
Effects of remifentanil preconditioning (RPC) on myocardial expression of Caspase-3 and Cav-3 and phosphorylation of Akt and STAT3 in N-acetylcysteine- (NAC-) treated diabetic rats. Streptozotocin-induced diabetic rats with or without NAC treatment in the presence or absence of wortmannin (Wort) and AG490 (AG) were subjected to myocardial ischemia/reperfusion (I/R) or RPC. (a–d) Representative Western blot of cleaved Caspase-3 compared with GADPH (a), Cav-3 compared with GADPH (b), p-Akt (ser^473^) compared with total Akt (c), and p-STAT3 (Tyr^705^) compared with total STAT3 (d). Differences were determined by one-way analysis of variance (ANOVA) followed by Tukey's test. ^∗^*P* < 0.05 and ^∗∗^*P* < 0.01 vs. the I/R group; ^#^*P* < 0.05 vs. the I/R+NAC group; and ^$^*P* < 0.05 vs. the NAC+RPC group.

We then examined the effects of RPC on myocardial Caspase-3, Cav-3 expression, and Akt and STAT3 phosphorylation in NAC-treated diabetic rats with or without treatment with wortmannin or AG490. As shown in Figure 6, RPC did not alter Caspase-3 cleavage, Cav-3 expression, phosphorylated Akt at ser^473^, and phosphorylated STAT-3 at Tyr^705^ in diabetic rats sustaining myocardial I/R. NAC treatment significantly decreased Caspase-3 cleavage and increased Cav-3 expression, phosphorylated Akt at ser^473^, and phosphorylated STAT-3 at Tyr^705^; all these alterations induced by NAC treatment were further enhanced by RPC. Either PI3K/Akt inhibitor wortmannin or the JAK2/STAT3 inhibitor AG490 abolished RPC-induced attenuation of Caspase-3 cleavage in NAC-treated diabetic rats undergoing myocardial I/R (Figure 6(a)). Additionally, wortmannin not only blocked Akt phosphorylation but also depressed STAT3 phosphorylation induced by NAC and RPC in diabetic rats (Figures 6(c) and 6(d)). In contrast, AG490 also inhibited NAC- and RPC-induced Akt and STAT3 phosphorylation. These indicate that cross talk exists between PI3K/Akt and JAk2/STAT3 signaling during RPC-mediated cardioprotection in NAC-treated diabetic rats. Of note, both wortmannin and AG490 had no effects on cardiac Cav-3 expression (Figure 6(b)), suggesting that Cav-3 may be an upstream signaling molecule of PI3K/Akt and JAk2/STAT3 signaling pathways.

### 3.6. Cardiac-Specific Knockdown of Cav-3 Expression Abolished RPC Cardioprotection in NAC-Treated Diabetic Rats

**Figure 7:**
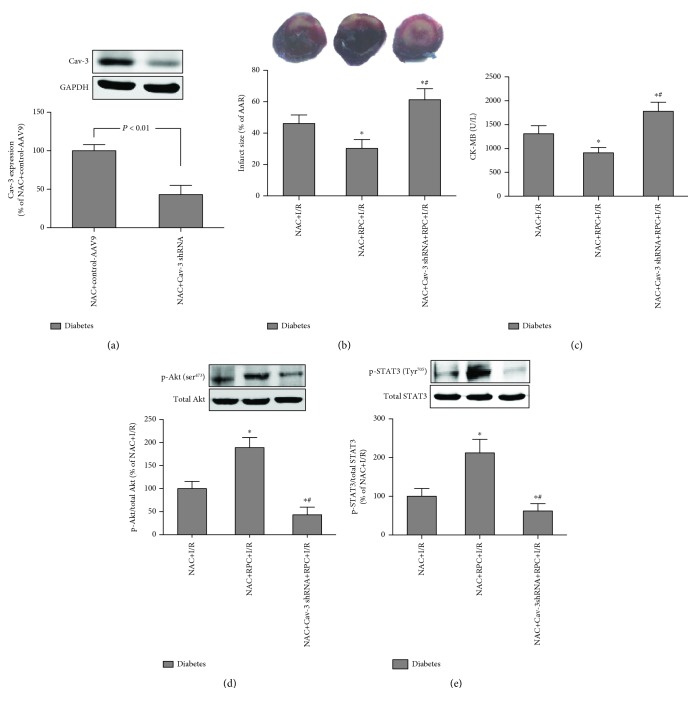
Cardiac-specific knockdown of Cav-3 expression abolished RPC cardioprotection in NAC-treated diabetic rats. NAC-treated diabetic rats were transfected with AAV9-Cav-3 shRNA for 3 weeks; then, the rats were subjected to myocardial I/R. (a–e) Representative Western blot of Cav-3 compared with GADPH (a), infarct size (b), plasma CK-MB (c), p-Akt (ser^473^) compared with total Akt (d), and p-STAT3 (Tyr^705^) compared with total STAT3 (e). All the results are expressed as mean ± S.D., *n* = 7. Differences were determined by one-way analysis of variance (ANOVA) followed by Tukey's test. ^∗^*P* < 0.05 vs. the diabetes+NAC+I/R group; ^#^*P* < 0.05 vs. the diabetes+NAC+RPC group.

To further explore the role of Cav-3 in RPC cardioprotection in NAC-treated diabetic rats and its relative roles in PI3K/Akt and JAk2/STAT3 signaling, the rats were either administrated with control-AAV9 or Cav-3 shRNA-AAV9. As shown in Figure 7(a), myocardial Cav-3 expression was significantly reduced in the rats transfected with Cav-3 shRNA-AAV9 as compared with control. This reduction of Cav-3 expression abolished the attenuation of the infarct size and CK-MB by NAC and RPC in diabetic rats (Figures 7(b) and 7(c)), indicating that Cav-3 is necessary for the restoration of RPC cardioprotection in NAC-treated diabetic rats. We then examined the effects of Cav-3 knockdown on Akt and STAT3 phosphorylation in NAC-treated diabetic rats. As shown in Figures 7(d) and 7(e), both Akt and STAT3 phosphorylation induced by RPC were significantly attenuated by Cav-3 shRNA-AAV9, indicating that Cav-3 are required for Akt and STAT3 activation.

### 3.7. NAC-Restored RPC Protection against Cardiomyocyte H/R Injury under HG Conditions In Vitro Involves Caveolae-Modulated Akt and STAT3 Activation

**Figure 8:**
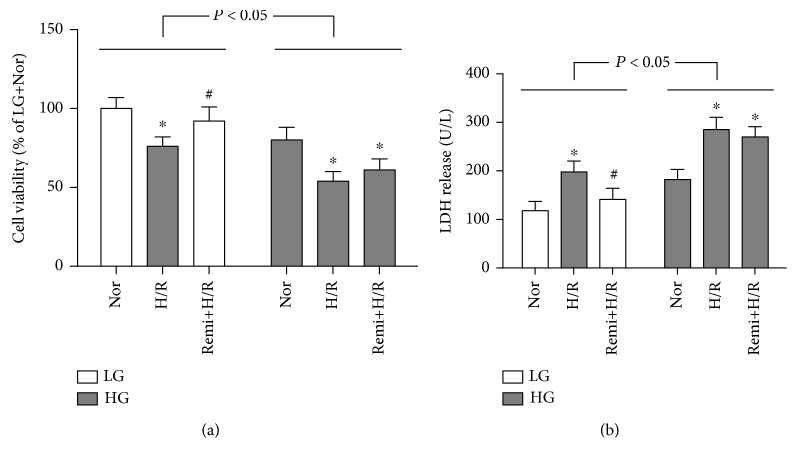
Remifentanil treatment attenuated posthypoxic cardiomyocyte injury under LG but not in HG conditions. Isolated rat cardiomyocytes were exposed to low glucose (LG, 5.5 mmol/L) or high glucose (HG, 25 mmol/L) with or without treatment of remifentanil (Remi, 2.5 *μ*M) for 36 h, then subjected to 4 hours of hypoxia followed by 4 hours of reoxygenation (H/R). Cell viability (a) and LDH release (b). All the results are expressed as means ± S.D., *n* = 7. Differences were analyzed by two-way repeated-measures ANOVA followed by Bonferroni's post hoc test. ^∗^*P* < 0.05 vs. the corresponding sham group; ^#^*P* < 0.05 vs. the corresponding I/R group.

**Figure 9:**
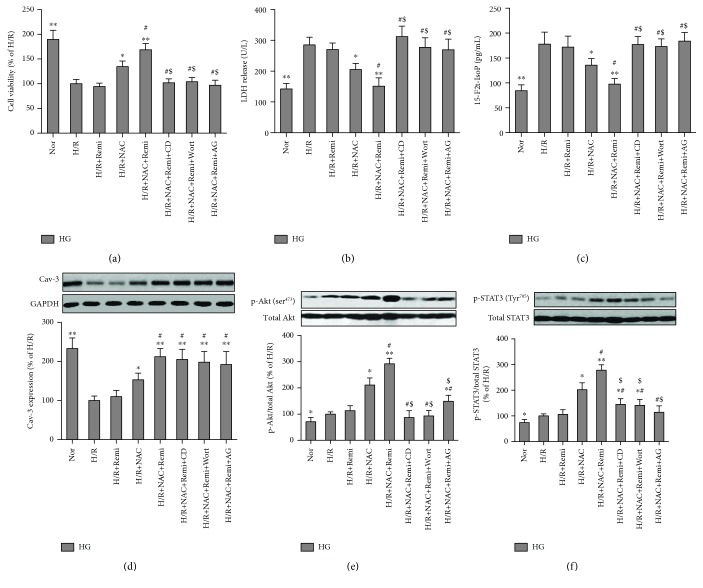
N-Acetylcysteine- (NAC-) restored remifentanil protection against HG and H/R-induced injury involves caveolae-modulated Akt and STAT3 activation in isolated cardiomyocytes. Isolated rat cardiomyocytes were exposed to high glucose (HG, 25 mmol/L) with or without treatment of methyl-*β*-cyclodextrin (CD, 10 *μ*M), wortmannin (Wort, 100 nM), AG490 (AG, 50 *μ*M), or combination of remifentanil (Remi, 2.5 *μ*M) and NAC (1 mmol/L) for 36 h, then subjected to 4 hours of hypoxia followed by 4 hours of reoxygenation (H/R). Cell viability (a), LDH release (b), 15-F2t-isoprostane (15-F2t-IsoP) (c), representative Western blot of Cav-3 compared with GADPH (d), p-Akt (ser^473^) compared with total Akt (e), and p-STAT3 (Tyr^705^) compared with total STAT3 (f). All these results are expressed as mean ± S.D., *n* = 7. Differences were determined by one-way ANOVA followed by Tukey's test. ^∗^*P* < 0.05 and ^∗∗^*P* < 0.01 vs. the HG+H/R group; ^#^*P* < 0.05 vs. the HG+H/R+NAC group; and ^$^*P* < 0.05 vs. the HG+H/R+NAC+Remi group.

We also explored the treatment effects of RPC in isolated cardiomyocytes under low glucose (LG) or high glucose (HG) conditions. As shown in Figures 8(a) and 8(b), HG significantly decreased cell viability and increased LDH release as compared with that in LG condition. H/R markedly decreased cell viability and increased LDH release as compared with the corresponding normoxic groups. Remifentanil administration significantly attenuated the decrease of cell viability and increase of LDH release by H/R stimulation in LG conditions but not in HG conditions (Figure 8). We then tested the beneficial effects and mechanisms of NAC on RPC in isolated cardiomyocytes exposed to HG and H/R. As shown in Figures 9(a)–9(c), H/R stimulation significantly decreased cell viability and increased LDH release and 15-F2t-IsoP productions in isolated primary cardiomyocytes under HG conditions as compared with normoxic groups and these alterations were not affected by remifentanil administration. However, remifentanil further enhanced NAC-induced improvements in posthypoxic cell viability and further reduced LDH release and 15-F2t-IsoP in cardiomyocytes (Figures 9(a)–9(c)). These protective effects were compromised by the disruption of caveolae by methyl-*β*-cyclodextrin (CD, a disrupter of cholesterol-rich caveolae [34]) or concomitant treatment with either wortmannin or AG490 (Figures 9(a)–9(c)), though CD, wortmannin, or AG490 alone had no significant effects on cell viability, LDH release, and 15-F2t-IsoP production in isolated primary cardiomyocytes under HG conditions (data not shown). These indicate that NAC treatment-mediated restoration of remifentanil cardiac protection under HG conditions requires normal caveola function and intact PI3K/Akt and JAK2/STAT3 signaling.

We then determined Cav-3 expression and phosphorylation of Akt and STAT3, as well as their association/interaction in isolated cardiomyocytes under HG and H/R conditions. As shown in Figures 9(d)–9(f), H/R stimulation significantly decreased Cav-3 expression and increased phosphorylation of Akt (ser^473^) and STAT3 (Tyr^705^) as compared with the normoxia (Nor) group, which were not altered by remifentanil administration under HG. Treatment with NAC increased Cav-3 expression and phosphorylation of Akt and STAT3, which were further enhanced by remifentanil. Further, the augmentation of Cav-3 expression in NAC- and remifentanil-treated cardiomyocytes exposed to HG and H/R was not affected by the administration with wortmannin or AG. In contrast, both Akt and STAT3 phosphorylation were significantly attenuated by caveola disruption with CD, indicating that caveolae are required for Akt and STAT3 activation but not vice versa. Of note, CD, always served as an agent of cholesterol depletion, disrupts the structure of lipid rafts and caveolae by reducing membrane-free cholesterol, which results in distorted caveola morphology and redistribution of the Cav-3 protein [39] and subsequently leads to altered cell signaling and function [40]. In the present study, we treated the isolated cardiomyocytes with CD for 1 h at the dose of 10 *μ*mol/L [34] and this dosage could disrupt caveola function without affecting Cav-3 expression in NAC- and RPC-treated cardiomyocytes exposed to HG and H/R (Figure 9(d)). In the preliminary study, we also treated the isolated cardiomyocytes with CD at a high dose of 5 mmol/L [40], but this dosage slightly decreased Cav-3 expression (data not shown). Furthermore, the inhibitors wortmannin and AG could inhibit Akt and STAT3 phosphorylation of each other in NAC- and remifentanil-treated cardiomyocytes exposed to HG and H/R (Figures 9(e) and 9(f)), providing further evidence to demonstrate the cross talk between Akt and STAT3 signaling [41].

### 3.8. NAC-Mediated Restoration of RPC Protection against HG and H/R-Induced Cellular Injury Requires Intact Cav-3-Modulated PI3K/Akt and JAK2/STAT3 Signaling in Cultured H9C2 Cells

**Figure 10:**
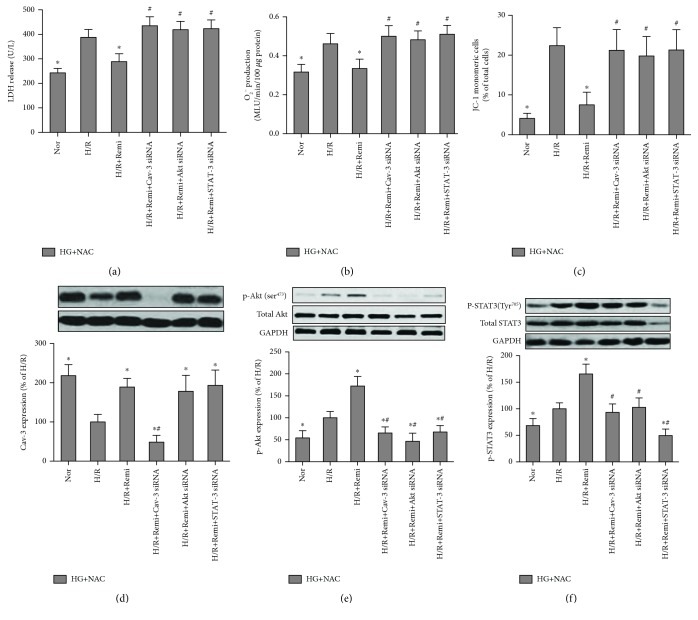
N-Acetylcysteine- (NAC-) preserved remifentanil protection against HG and H/R-induced injury requires intact Cav-3-modulated PI3K/Akt and JAK2/STAT3 signaling in H9C2 cells. H9C2 cells transfected with or without Cav-3 siRNA, Akt siRNA, or STAT3 siRNA were treated with or without remifentanil (Remi, 2.5 *μ*M) treatment in the presence of NAC (1 mmol/L) and high glucose (HG) with normoxia (Nor) for 36 h, then subjected to 4 hours of hypoxia followed by 4 hours of reoxygenation (H/R). LDH release (a), O_2_^−^ production (b), percentage of JC-1 monomeric cells in total cells (c), and Cav-3 (d), Akt (e), and STAT3 (f) expression and their phosphorylation. All the results are expressed as mean ± S.D., *n* = 7. Differences were determined by one-way analysis of variance (ANOVA) followed by Tukey's test. ^∗^*P* < 0.05 vs. the HG+NAC+H/R group; ^#^*P* < 0.05 vs. the HG+NAC+H/R+Remi group.

To further investigate the roles of Cav-3, PI3K/Akt, and JAK2/STAT3 signaling in the beneficial effects of remifentanil in NAC-treated conditions in *in vitro* studies, the H9C2 cells were transfected with Cav-3 siRNA, Akt siRNA, or STAT3 siRNA. As shown in Figures 10(a)–10(c), remifentanil significantly attenuated the H/R-induced increase of LDH release and O_2_^−^ production in NAC-treated cells cultured in HG conditions and reduced the percentage of JC-1 monomeric cells, an indicator of the loss of MPP, which was used to evaluate mitochondrial damage. However, these effects were abolished, respectively, by Cav-3 siRNA, Akt siRNA, or STAT3 siRNA. Remifentanil in combination with NAC reverted H/R-induced reduction of Cav-3 expression while further enhancing H/R-induced increases in Akt and STAT3 phosphorylation (Figures 10(d)–10(f)). H9C2 cells treated with rat-specific Cav-3 siRNA exhibited about 60% reduction in Cav-3 expression during both LG and HG conditions (data not shown). This reduction of Cav-3 expression resulted in the attenuation of remifentanil-induced Akt and STAT3 phosphorylation in NAC-treated cells cultured in HG conditions, indicating that Cav-3 is necessary for Akt and STAT3 activation. The expression of total Akt and total STAT3 was markedly reduced by transfection with Akt siRNA or STAT3 siRNA in H9C2 cells. Neither Akt siRNA nor STAT3 siRNA had significant effects on Cav-3 expression. In contrast, either Akt siRNA or STAT3 siRNA inhibited Akt and STAT3 phosphorylation (Figures 10(e) and 10(f)).

## 4. Discussion

In the present study, we have demonstrated that the compromised RPC protection against myocardial I/R injury in diabetes is associated with hyperglycemia-induced excessive oxidative stress, caveola dysfunction, and altered Cav-3 expression, which results in impaired PI3K/Akt and JAK2/STAT3 signaling. Treatment with the antioxidant NAC attenuated cardiac dysfunction and restored RPC cardioprotection through improving Cav-3-modulated PI3K/Akt and JAK2/STAT3 signaling. To our knowledge, this is the first study to explore the relative roles of Cav-3, PI3K/Akt, and JAK2/STAT3 signaling in RPC in diabetes, as well as the effectiveness of antioxidant treatment with NAC to restore RPC cardioprotection in diabetes.

Ischemic (pre, post, and remote) conditioning strategies and pharmacological treatments are well described to enhance the ability of the heart to withstand a prolonged I/R insult, but their translation from experimental to clinical studies for improving patient outcomes has been both challenging and disappointing due to the complex disorders (e.g., diabetes and hyperglycemia) in human [42, 43]. Opioid preconditioning confers acute and delayed cardioprotection via opioid receptors (OR), similar to ischemic preconditioning [13, 44]. Given that the presence of *δ* and *κ* but not *μ*-OR has been demonstrated in the myocardium [44, 45], it is not surprising that both the selective agonist of *δ*-OR and *κ*-OR confers cardioprotection in many species [46, 47]. Although remifentanil is predominantly a *μ*-OR agonist, it reduces myocardial infarction in normal rats [12, 14] irrespective of whether it is used for preconditioning, postconditioning, or continuous infusion during ischemia and reperfusion [14]. This cardioprotection induced by remifentanil may be attributable to the weak activity of *μ*-OR on the *δ*-OR and *κ*-OR [48] or possibly receptor cross talk between the *μ*-OR and *δ*-OR [49]. However, under hyperglycemic conditions, RPC-induced protection against myocardial I/R injury is compromised [10, 15]. These were well demonstrated by our present study that RPC significantly attenuated myocardial I/R injury in control but not in diabetic rats, but it is still unknown whether diabetes affects the myocardial *δ* and *κ*-OR status or the activity of remifentanil on the *δ* and *κ*-OR, which may impair RPC in diabetes.

The pathogenesis of myocardial I/R injury in diabetes is complicated, but evidence indicates the involvement of oxidative stress induced by hyperglycemia [2, 37, 50]. We have shown that excessive oxidative stress, as demonstrated by elevated production of plasma and cardiac 15-F2t-isoprostane (a specific marker of oxidative injury [33]) at baseline, may have contributed to the decreased tolerance of the diabetic heart to myocardial I/R injury [24]. We speculated that the excessive oxidative stress induced by hyperglycemia was also attributable to the compromised cardioprotection of RPC. Indeed, after the treatment of NAC, a thiol-containing radical scavenger and a glutathione precursor, RPC attenuated myocardial I/R-induced postischemic cardiac dysfunction, infarct size, and cardiomyocyte apoptosis in diabetes, accompanied with decreased levels of plasma CK-MB, plasma 15-F2t-IsoP, and cardiac 15-F2t-IsoP. Further, remifentanil administration significantly reduced HG and H/R-induced LDH release and 15-F2t-IsoP production and increased cell viability in isolated cardiomyocytes in the presence of NAC. Thus, the compromised cardioprotection of RPC in diabetes may be in part explained by the excessive oxidative stress status induced by hyperglycemia and antioxidant treatment may be a useful therapy to preserve the effectiveness of RPC-mediated cardioprotection in diabetes.

Diabetes is known to induce fundamental alterations in cellular signaling cascades that affect the development of I/R injury per se and responses to cardioprotective interventions [43]. It is well established that the activation of PI3K/Akt and JAK2/STAT3 signaling is involved in cardiac protection against I/R injury [16, 17, 51] and also plays an important role in opioid-induced cardioprotection [12, 15, 18, 19]. However, both signaling pathways have been shown to be impaired in diabetic conditions [6, 25], which may render diabetic hearts more susceptible to I/R injury and less sensitive to opioid conditioning. This is well demonstrated in the present study. Our results indicate that the involvement of hyperglycemia-induced oxidative stress and impaired PI3K/Akt and JAK2/STAT3 signaling in compromising the cardioprotection of RPC and suppressing oxidative stress with NAC may attenuate myocardial I/R injury and preserve RPC-induced cardioprotection by restoring PI3K/Akt and JAK2/STAT3 signaling in diabetes. It is noteworthy that selective inhibition of these two signaling pathways abolished RPC cardioprotection in NAC-treated diabetic rats, accompanied with increased oxidative injury. This oxidative injury may be derived from myocardial I/R injury, as myocardial I/R may result in mitochondrial dysfunction with oxidative damage [52].

Although the precise mechanisms by which hyperglycemia-induced inhibition of Akt and STAT3 activation compromise RPC-induced cardioprotection are not fully understood, the reduced expression of Cav-3 during hyperglycemia in diabetes might be a major factor [25, 26]. The diabetes-induced decrease of Cav-3 expression may lead to caveola dysfunction, which plays a vital role in affecting PI3K/Akt and JAK2/STAT3 signaling in diabetic hearts. This is well supported by our findings that Cav-3 knockdown or disruption of caveola function with methyl-*β*-cyclodextrin inhibited both Akt and STAT3 phosphorylation in *in vivo* and *in vitro* studies. Neither the putative PI3K/Akt inhibitor wortmannin [6] nor the JAK2/STAT3 inhibitor AG490 [18] had significant effects on Cav-3 expression, suggesting that Cav-3 may be an upstream signaling molecule of PI3K/Akt and JAk2/STAT3 signaling pathways. Additionally, wortmannin not only blocked Akt phosphorylation but also depressed STAT3 phosphorylation induced by NAC and RPC. In contrast, AG490 also inhibited NAC- and RPC-induced Akt and STAT3 phosphorylation. Similar effects were shown in H9C2 cells transfected with Akt siRNA and STAT3 siRNA. These findings provide further evidence to demonstrate the cross talk between PI3K/Akt and JAK2/STAT3 signaling [41] and suggest that these two signaling pathways are modulated by Cav-3. To our knowledge, this is the first study to provide direct evidence to demonstrate the relationships among Akt, STAT3, and Cav-3 in cardiomyocytes and their link to hyperglycemia-induced oxidative stress.

Cav-3 is a major isoform of the caveolin family proteins to form cardiomyocyte caveolae [20], serving as a platform to enrich cardioprotective signaling molecules, including PI3K/Akt [53] and JAK/STAT [54]. It has been reported that both caveolae and caveolin-3 are critical for retaining myocardial tolerance to I/R injury [21, 55] and Cav-3 expression is also required for opioid induced cardioprotection [23]. Therefore, any alteration of Cav-3 expression in diabetes may be implicated in the compromised RPC-mediated cardioprotection, and thus, restoring Cav-3 expression should be beneficial to preserve the effectiveness of RPC in diabetes. In the present study, decreased Cav-3 expression was detected in hearts from 5-week duration of diabetes, which is consistent with our previous studies [25, 26]. A very recent study showed that Cav-3 could be degraded by a ubiquitin-proteasome system in cardiomyocytes [56] and oxidative stress may lead to the activation of a ubiquitin-proteasome system [57]. Thus, hyperglycemia-induced oxidative stress may have increased Cav-3 degradation through activating a ubiquitin-proteasome system in the diabetic hearts or in high-glucose-stimulated cardiomyocytes, though further study is merited to test this hypothesis. Additionally, myocardial I/R attenuated cardiac Cav-3 expression in control rats and further decreased Cav-3 expression in diabetic rats. NAC treatment induced restoration of Cav-3 expression, attenuated myocardial I/R injury, and restored RPC-induced cardioprotection in *in vivo* studies, and similar effects have been observed in *in vitro* studies. However, the beneficial effects of NAC in diabetic rats, isolated cardiomyocytes, and H9C2 cells were abolished by cardiac-specific knockdown of Cav-3 expression with Cav-3 shRNA-AAV9, concomitant treatment with inhibitors methyl-*β*-cyclodextrin, wortmannin, and AG490 or transfection with Cav-3 siRNA, Akt siRNA, and STAT3 siRNA, suggesting that the beneficial effects of NAC in restoring RPC-induced cardioprotection in diabetic hearts are achieved through Cav-3-modulated Akt and STAT3 signaling. These findings may have clinical implications in developing therapies to incorporate alternative antioxidant treatment and to improve Cav-3/Akt and/or Cav-3/STAT3 signaling in combating myocardial I/R injury and preserving RPC-induced cardioprotection in diabetes.

**Figure 11:**
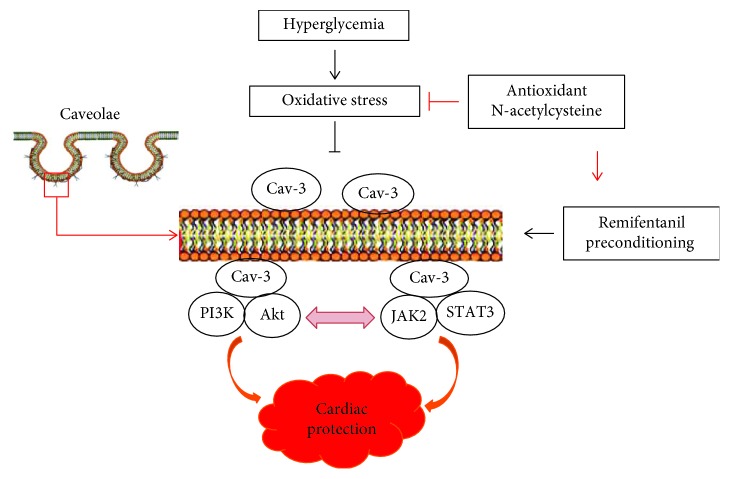
Schematic depicting that hyperglycemia-induced oxidative stress compromises RPC cardioprotection in diabetes by impairing Cav-3-modulated PI3K/Akt and JAk2/STAT3 signaling. Antioxidant N-acetylcysteine preserves RPC-induced cardioprotection by improving Cav-3-dependent Akt and STAT3 activation and by facilitating the cross talk between PI3K/Akt and JAK2/STAT3 signaling pathways in diabetes. Solid arrows depict stimulation, while transverse “T” shape indicates inhibition.

In summary, the current results demonstrate that hyperglycemia-induced oxidative stress is involved in the impaired Cav-3-modulated PI3K/Akt and JAk2/STAT3 signaling, which ultimately compromises RPC cardioprotection in diabetes (Figure 11). Antioxidant NAC preserves RPC-induced cardioprotection by improving Cav-3-dependent Akt and STAT3 activation and by facilitating the cross talk between PI3K/Akt and JAK2/STAT3 signaling pathways in diabetes (Figure 11). Our data suggest that antioxidant treatment and/or improvement of caveolae-modulated signaling may be useful approaches for attenuating myocardial I/R injury and preserving the effectiveness of therapeutic strategies, such as ischemic and opioid conditioning.

## Figures and Tables

**Table 1:** General characteristics of diabetic rats at the end of the treatment period.

	C	D	D+NAC
Water intake (mL/kg/day)	127.8 ± 21.5	848.7±36.9^∗∗^	476.4±34.4^∗∗^^,#^
Food consumption (g/kg/day)	72.3 ± 10.6	186.8±18.5^∗∗^	126.6±15.8^∗∗^^,#^
Body weight (g)	477.5 ± 24.7	295.2.7 ± 18.8^∗^	381.3 ± 20.6^∗^^,#^
Plasma glucose (mM)	6.76 ± 0.72	28.67±6.55^∗∗^	24.61±5.51^∗∗^
Plasma insulin (mU/L)	39.76 ± 8.47	13.56±4.39^∗∗^	14.52±4.97^∗∗^
Plasma triglycerides (mM)	0.71 ± 0.21	6.67±1.81^∗∗^	3.32 ± 0.63^∗^^,#^

Control (C) or STZ-induced diabetic (D) rats were treated with or without N-acetylcysteine (1.5 g/kg/day, D+NAC) by oral gavage for four weeks. All the results are expressed as mean ± S.D., *n* = 8. Differences in general characteristics were determined by one-way analysis of variance (ANOVA) followed by Tukey's test. ^∗^*P* < 0.05 and ^∗∗^*P* < 0.01 vs. the C group; ^#^*P* < 0.05 vs. the D group.

**Table 2:** Hemodynamics at baseline and after 2 h of reperfusion.

	HR (bpm)	LVSP (mmHg)	+dp/dt (mmHg/s)	-dp/dt (mmHg/s)
Baseline (10 min before ischemia)				
C	371 ± 17	122 ± 8	6750 ± 652	4908 ± 634
C+RPC	374 ± 15	123 ± 6	6785 ± 660	4952 ± 645
D	327 ± 12^#^	107 ± 7^#^	5220 ± 513^#^	3724 ± 516^#^
D+RPC	329 ± 11^#^	106 ± 7^#^	5240 ± 524^#^	3755 ± 521^#^
D+NAC	343 ± 14^#^	117 ± 8	6325 ± 455^$^	4718 ± 503^$^
D+NAC+RPC	342 ± 13^#^	116 ± 6	6330 ± 462^$^	4789 ± 524^$^
After 2 h of reperfusion				
C	324 ± 10^∗^	101 ± 6^∗^	4658 ± 486^∗^	3356 ± 462^∗^
C+RPC	352 ± 14^#^	117 ± 7^#^	5884 ± 508^#^	4458 ± 508^#^
D	271 ± 12^∗^^,#^	83 ± 8^∗^^,#^	3258 ± 438^∗^^,#^	2220 ± 365^∗^^,#^
D+RPC	267 ± 17^∗^^,#^	85 ± 7^∗^^,#^	3116 ± 449^∗^^,#^	2332 ± 408^∗^^,#^
D+NAC	298 ± 15^∗^^,$^	97 ± 5^∗^^,$^	4316 ± 455^∗^^,$^	3230 ± 353^∗^^,$^
D+NAC+RPC	327 ± 13^$,&^	110 ± 6^$,&^	5530 ± 523^$,&^	4451 ± 420^$&^

Control (C), diabetic (D), and N-acetylcysteine-treated diabetic rats (D+NAC, 1.5 g/kg/day) were subjected to myocardial ischemia/reperfusion or remifentanil preconditioning (RPC). The heart rate (HR), left ventricular systolic pressure (LVSP), maximum rate of increase of left ventricular developed pressure (+dp/dt), and maximum rate of decrease of left ventricular developed pressure (-dp/dt) were recorded at 10 min before ischemia (baseline) and 2 h after reperfusion. All the results are expressed as mean ± S.D., *n* = 8. Differences in hemodynamics at baseline and after 2 h of reperfusion were analyzed by two-way repeated-measures ANOVA followed by Bonferroni's post hoc test. ^∗^*P* < 0.05 vs. their corresponding baseline; ^#^*P* < 0.05 vs. their corresponding C group; ^$^*P* < 0.05 vs. their corresponding D group; and ^&^*P* < 0.05 vs. their corresponding D+NAC group.

## Data Availability

The data used to support the findings of this study are included within the article.
